# Crystals unveiled: looking at urine can be quite useful

**DOI:** 10.1590/2175-8239-JBN-2023-0160en

**Published:** 2024-01-22

**Authors:** Filipa Ferreira, Núria Paulo, Altin Ndrio

**Affiliations:** 1 Centro Hospitalar Universitário de São João, Departamento de Nefrologia, Porto, Portugal.; 2 Centro Hospitalar Universitário de São João, Departamento de Patologia Clínica, Porto, Portugal.

A 72-year-old kidney transplant patient was admitted to the hospital due to constitutional symptoms that had been present for one month and diffuse nodular skin lesions. He had been transplanted for 2.5 years and was receiving maintenance immunosuppression with prednisolone, tacrolimus, and mycophenolate. He had recent contact with cats and chickens. A diagnosis of disseminated toxoplasmosis was made after PCR detection of *Toxoplasma gondii* in the blood and in the cutaneous lesions. After one month of sulfadiazine and pyrimethamine treatment, sulfadiazine crystals were identified on the urinary sediment. Drug-induced crystalluria can occur due to drug overdose, dehydration, or hypoalbuminemia and can lead to acute kidney injury due to tubular obstruction.

**Figure 1.**
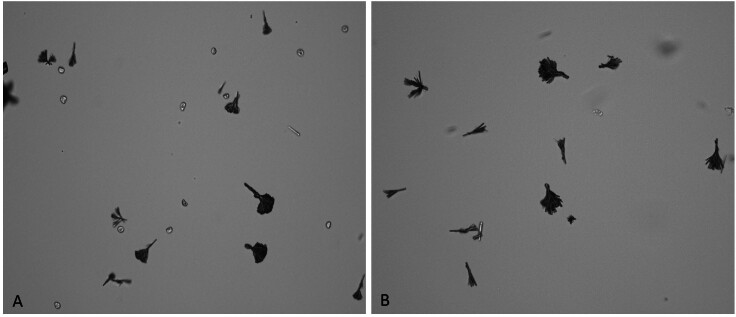
Images of urinary sediment showing typical forms of sulfadiazine crystals (A and B) and rare erythrocytes (A).
